# On the Use of MOFs and ALD Layers as Nanomembranes for the Enhancement of Gas Sensors Selectivity

**DOI:** 10.3390/nano9111552

**Published:** 2019-10-31

**Authors:** Matthieu Weber, Octavio Graniel, Sebastien Balme, Philippe Miele, Mikhael Bechelany

**Affiliations:** 1 Institut Européen des Membranes, IEM – UMR 5635, ENSCM, CNRS, University of Montpellier, Place Eugène Bataillon, 34095 Montpellier Cedex 5, France; matthieu.weber@umontpellier.fr (M.W.); ograniel@gmail.com (O.G.); sebastien.balme@umontpellier.fr (S.B.); philippe.miele@umontpellier.fr (P.M.); 2 Institut Universitaire de France (IUF), 1 Rue Descartes, 75231 Paris, France

**Keywords:** gas sensors, metal organic frameworks, atomic layer deposition, selectivity

## Abstract

Improving the selectivity of gas sensors is crucial for their further development. One effective route to enhance this key property of sensors is the use of selective nanomembrane materials. This work aims to present how metal-organic frameworks (MOFs) and thin films prepared by atomic layer deposition (ALD) can be applied as nanomembranes to separate different gases, and hence improve the selectivity of gas sensing devices. First, the fundamentals of the mechanisms and configuration of gas sensors will be given. A selected list of studies will then be presented to illustrate how MOFs and ALD materials can be implemented as nanomembranes and how they can be implemented to improve the operational performance of gas sensing devices. This review comprehensively shows the benefits of these novel selective nanomaterials and opens prospects for the sensing community.

## 1. Introduction

The detection and measurement of gas concentrations are crucial for both the understanding and the monitoring of industrial and environmental processes. In recent years, the demand for gas detection and gas measurement tools has considerably increased, mainly due to safety, process and environmental control considerations [1].

In the field of gas detection, there is a distinction between analyzers (analytical instruments such as chromatographs and spectrometers) and chemical sensors. The analyzers allow for very precise measurements but are relatively complex systems associating different mechanical, chemical and electrical elements. Those devices are often expensive and energy-intensive, making them poorly suited for on-site measurements. In addition, these instruments often present long response times, due to the detection technique itself or to the need for sample preparation. The main advantage of these instruments is the possibility to obtain a complete and accurate analysis of the gas sample.

On the other hand, chemical sensors are usually based on simple systems. This type of sensor consists of a sensitive layer for the gas detection with which it interacts, and a transducer system transforming this chemical interaction into a measurable electrical signal. The main advantages of this type of sensor are their limited size and cost, their short response times and their low energy consumption. These key merits make them crucial instruments for on-site measurements and online process control. There are several families of chemical sensors that can be classified by the type of sensitive layer and the principle of transduction. For example, sensors can be based on catalytic or piezoelectric materials, but due to their good sensitivity and ease of fabrication using microelectronic manufacturing technologies, most of the sensors are based on semiconductor sensitive layers. The conductivity of a semiconductor material surface depends on the composition of the neighboring atmosphere, and the signal measured is therefore a simple variable resistor. The sensing mechanism of this type of sensor is directly linked to the gases’ adsorption at the surface of the material, altering its electrical conductivity. Semiconductor metal oxides (SMO) are the most commonly used sensing materials [2,3]. Besides, as they present excellent stability and benefit of low-cost fabrication processes, they are strong candidates for a wide range of sensing applications. Thus, despite their relative lack of selectivity, semiconductor-based gas sensors have been marketed since the 1960s and widely used [1,2,3,4,5,6]. In fact, metal oxide semiconductors have been the foremost materials applied to gas sensing because of their easy fabrication, good stability, nanostructure diversity, and high sensitivity to various gas molecules [1,2,3,4,5,6]. Yet, these types of sensors present relatively low selectivity, limiting their practical use, and more research is still required to increase the selectivity of sensors. 

Different strategies for the enhancement of sensing characteristics can be applied, and innovative nanomembrane materials can be efficiently used to improve gas sensing selectivity. Nanomembranes can be defined as materials with nanoscale thicknesses separating different media, and enabling the selective passage of certain species. Such materials have practical appeal for sensing applications, because their extremely small thicknesses and realistic pathways to manufacturing facilitate integration into devices. 

MOFs are a novel class of materials with high porosity, based on metallic nodes and organic linkers that have recently attracted considerable attention. These tunable materials present a large number of benefits such as uniform channels, high internal surface areas, sub-nanometer-sized cavities, and stability [7,8,9,10]. MOFs nanomaterials presenting defined and controllable porosity are promising candidates for a wide array of applications, including gas separation and sensing [11,12].

Atomic layer deposition (ALD) is a vapor phase deposition technique allowing the precise synthesis of ultrathin films of inorganic nanomaterials such as oxides [13,14,15,16], nitrides [17,18,19,20,21], and metals [22,23], with an extreme control over the thickness [24,25]. This technique can be applied to coat challenging 3D substrates such as complex nanostructures with a conformal and uniform layer of high quality material, a capability unique among the many film deposition techniques [25,26]. 

In this minireview, the fundamentals of the mechanisms and configuration of gas sensors will be given, and the use of MOFs and ALD films as nanomembranes towards the separation of different gases and the enhancement of gas sensor selectivity will be presented. Selected studies will be employed to illustrate how these nanomaterials can be implemented in gas sensing devices and how they can improve their performance.

## 2. Fundamentals of the Mechanisms and Configuration of Gas Sensors

### 2.1. Gas Sensing Mechanism of Metal Oxide-Based Gas Sensors

**Figure 1:**
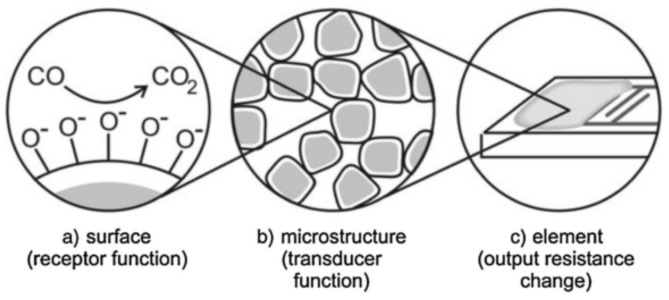
Receptor and transducer functions of a semiconductor gas sensor: (**a**) surface, providing the receptor material function, (**b**) the sensing layer microstructure, providing the transducer function, and (**c**) the element, allowing the detection of the modification in output resistance of the sensing nanolayer, here prepared on an interdigital microelectrode. Reprinted with permission from [30]. Copyright John Wiley and Sons, 2006.

Even though the working principle of SMO-based gas sensors is simple, the detection mechanism behind them is quite complex [27]. It is generally accepted that the sensing mechanism of SMO sensors is mainly governed by its receptor function and its transducer ability [28]. The transducer function is related to the ability of the oxide surface to react with the target gas, while the transducer function concerns the conversion of this chemical interaction into a measurable electrical signal [29]. Figure 1 shows the receptor and transducer functions [30].

**Figure 2:**
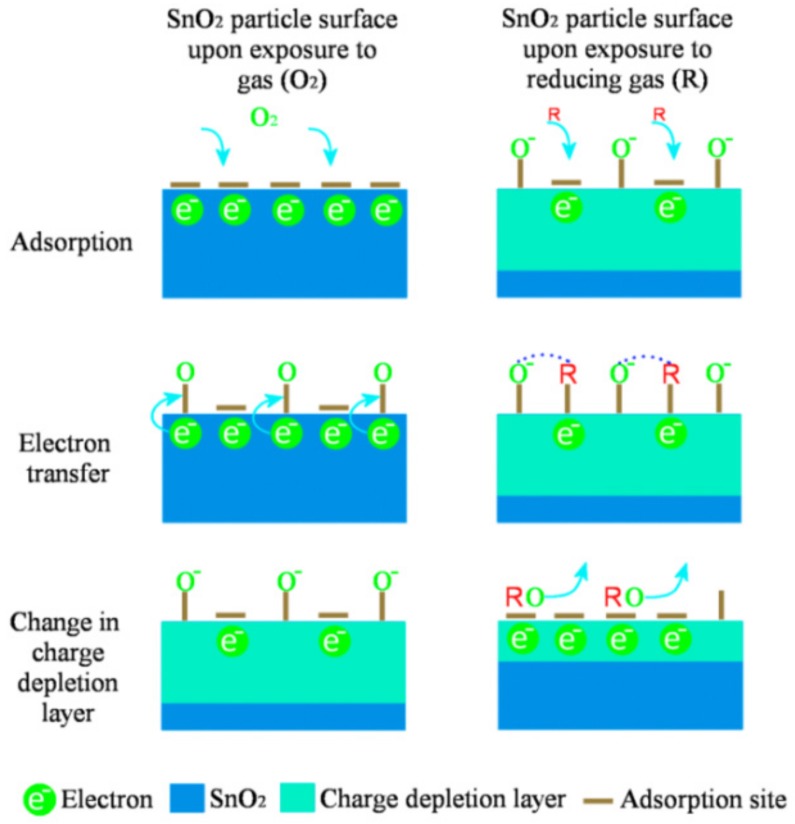
Schematics of the SnO_2_ sensing response towards oxidizing and reducing gases. Reprinted from [31].

For instance, when atmospheric oxygen is adsorbed on an n-type metal oxide like SnO_2_, a depletion layer at the surface of SnO_2_ is formed due to the withdrawal of electrons from the underlying SnO_2_ film by oxygen. Interaction of reducing agents with this electron-depleted region leads to a decrease in the band bending that is translated into an increase of conductivity whereas, in the case of oxidizing agents, a decrease in conductivity can be observed. Figure 2 presents a schematic representation of the SnO_2_ mechanism towards oxidizing and reducing agents.

Given that most metal oxide gas sensors are operated at temperatures between 150 °C and 450 °C, the dominant oxygen species adsorbed at the surface is $O^{-}$, while below 150 °C oxygen is ionosorbed predominantly as $O_{2}^{-}$. As shown in Figure 2, when SnO_2_ is exposed to a reducing gas like CO, the following reactions can take place:

(1) $$\mathrm{CO}+O_{\mathrm{ads}}^{-}\to {\mathrm{CO}}_{2}+e^{-}$$

and

(2) $$2\mathrm{CO}+O_{2,\mathrm{ads}}^{-}\to 2{\mathrm{CO}}_{2}+e^{-}$$

which decrease the amount of adsorbed oxygen and releases the surface-trapped electrons back to the SnO_2_ bulk [32].

### 2.2. Device Configuration of Metal Oxide-Based Gas Sensors

**Figure 3:**
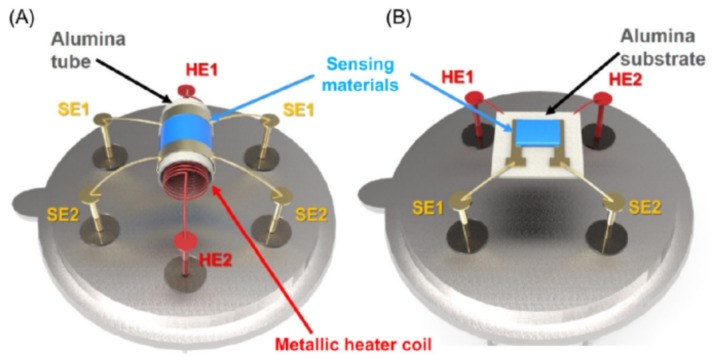
Schematic structure of (**A**) tube-type and (**B**) substrate-type oxide semiconductor sensors (SE, sensor electrode; HE, heater electrode). Reprinted with permission from [34]. Copyright Elsevier, 2019.

The majority of SMO-based gas sensors consist of three main parts: a sensitive layer, electrodes, and a heater [33]. The sensitive layer, which should react upon exposure to the gas of interest, is usually supported on alumina ceramics that can have a tubular or planar shape (Figure 3).

**Figure 4:**
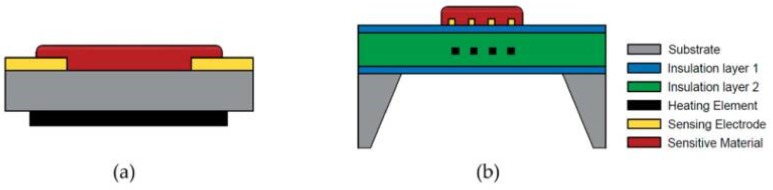
Schematic representations of (**a**) a common metal oxide gas sensor and (**b**) a micro-hotplate metal oxide gas sensor. Reprinted from [36].

The electrodes are used to transmit the output current flow to the terminal, and the heating element is used to warm up the sensitive layer so it can reach an optimum working temperature. As a material with high thermal stability, low thermal expansion coefficients, high mechanical strength, and cost effectiveness, alumina remains one of the best support materials available on the market. Recently, silicon wafers have also been used as substrates due to their compatibility with integrated circuits and the possibility to fabricate miniaturized gas sensors with them [35]. For comparison, the configuration of a traditional metal oxide gas sensor and a micro-electro-mechanical system (MEMS)-based sensor is shown in Figure 4. Unlike the classical configuration of metal oxide gas sensors, the heating element is integrated in a micro-hotplate that is sandwiched between two insulating layers (e.g., silicon oxide or silicon nitride) that protect the sensing material from the substrate and prevent catalytic reactions between the target gas and the heater material.

**Figure 5:**
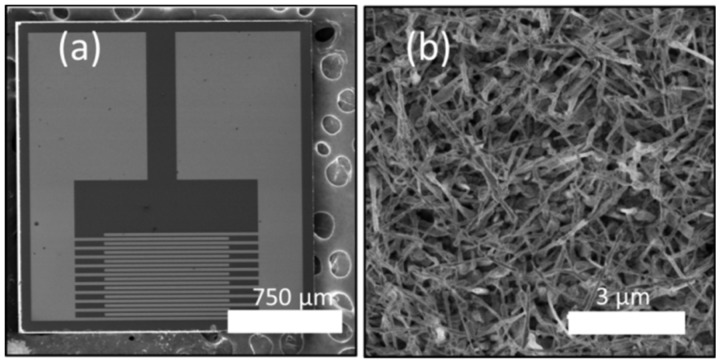
SEM images (top view) of (**a**) the gas sensing device and (**b**) network of the ZnO nanowires covering the sensor surface. Taken and adapted from [39] with permission from The Royal Society of Chemistry, 2019.

The physicochemical properties at the surface of the metal oxide define how the target gas is reaching the first atomic layers, and the sensing of gases can be achieved by different means, the main operating principle being the reversible modification of the electrical conductivity changed by the interaction between the metal oxide surface and the species in the direct gas environment. However, gas sensing can also be carried out by other means, for example, by monitoring changes in the optical or mass signals of the active layer [37,38]. Thanks to the fast development of the microelectronic industry, the large majority of metal oxide gas sensors are now miniaturized, and often based on nanostructures. For example, our group recently worked on the tuning of millimeter scaled hydrogen sensors based on semiconductor ZnO nanowires (see Figure 5), by making use of the change in resistivity on exposure to the relevant gas.

## 3. MOFs as Nanomembranes to Improve the Selectivity of Gas Sensing Devices

There is a pressing need for sensing devices with improved selectivity, rather than sensors with enhanced sensitivity, particularly for gas sensing applications. Metal organic frameworks (MOFs) constitute a novel class of crystalline materials that are formed through the self-assembly of metal cations (nodes) and organic ligands (linkers) [40,41,42]. Their diverse structural topologies, unprecedented porosity, and versatile chemical functionalities (host-guest interactions) have made them attractive for applications such as gas storage and separation [43], fabrication of luminescent materials [44], drug delivery [45], and catalysis [46]. Recently, the potential of MOFs to improve the selectivity in gas sensing elements has been explored [47,48,49,50]. Among the possible mechanisms of gas selectivity, the most straightforward is the size-exclusion effect (molecular sieving) where only analytes that are smaller than the pore channels of the MOF can be adsorbed, while larger analytes cannot pass through. The size and aperture of the pores can be fine-tuned by carefully selecting the appropriate metal ion clusters and organic linker sizes and shapes, controlling the catenation (i.e., interpenetration or interweaving of identical frameworks), and taking into account the directional mobility of the linkers [51].

In addition, the selectivity of MOFs towards specific analytes can occur through chemical functionalities that provide active sites for hydrogen bonding, van der Waals interactions, π-π interactions, and formation of coordinate covalent (dative) bonds. This type of selectivity can be achieved by incorporating the desired functionality directly into the synthesis process. For instance, it has been shown that NH_2_-MIL-101(Al) presents a higher affinity for CO_2_ than CH_4_ thanks to its amine moieties that induce van der Waals interactions between adsorbate and adsorbent [52]. However, the direct synthesis of highly functionalized MOFs has been restricted to just a few functional groups. This restriction is imposed by the thermal instability of the ligands, problematic solubility, and tendency to coordinate metal ions under solvothermal conditions. Luckily, alternative approaches that involve post synthetic methods have been developed [53]. Instead of introducing the functional groups directly into the organic ligands, post synthetic methods focus on the chemical modification of the fabricated MOF. For example, a reagent can be used to modify the organic linkers of MOFs to covalently bond a new functional group. Similarly, a ligand can be added to form a dative bond with unsaturated metal sites in the MOF, or a metal ion can be added to the MOF to form a dative bond with the organic linkers. In this section, the use of MOFs as nanomembranes to improve the selectivity in gas sensing applications will be divided into molecular sieving related effects, interaction with specific adsorption sites, and other effects.

### 3.1. Molecular Sieving Effect

**Figure 6:**
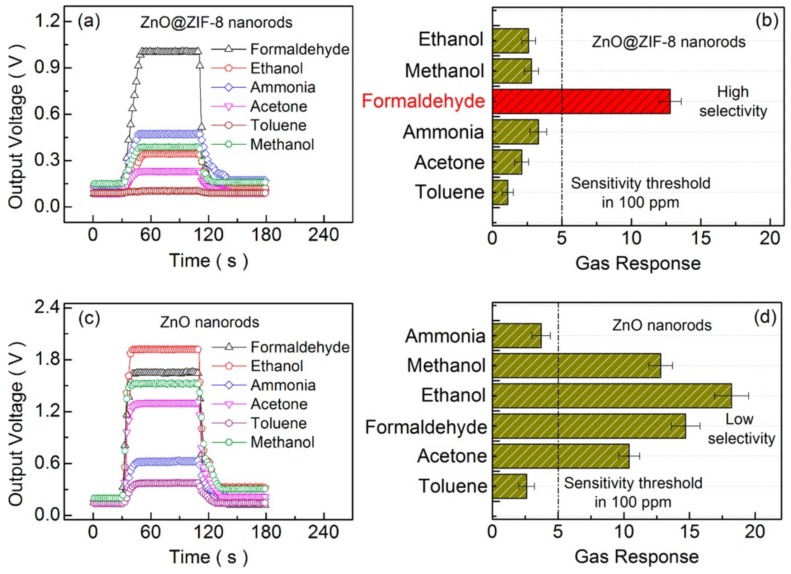
(**a**,**b**) Transient curves of the gas response and the selectivity of the ZnO@ZIF−8 nanorods sensor to 100 ppm of various VOCs at 300 °C, and (**c**,**d**) transient curves of gas sensing and the selectivity of the nanosensor to 100 ppm of various VOCs at 300 °C. Reprinted with permission from [58]. Copyright American Chemical Society, 2016.

An increasing number of studies have focused on the size-selective sieving properties of MOFs to take up specific guest molecules in gas sensing devices. For instance, the chemically robust and thermally stable zinc-based zeolite imidazole framework (ZIF-8) has been recently used as a membrane in semiconductor metal oxides (SMOs)-based sensors for selective gas sensing [54,55,56,57,58,59,60,61]. Owing to its large cavities (11.6 Å) and small pores apertures (3.4 Å), ZIF-8 allows the selective permeation of gas molecules smaller than the pores aperture while rejecting the penetration of larger gas molecules. In a first study, Drobek et al. fabricated ZnO nanowires by vapor deposition on a silicon-based interdigitated electrode (IDE) architecture, which were solvothermally converted to produce a thin ZIF-8 selective barrier [54]. The electrical resistance changes of ZIF-8@ZnO heterostructure and pristine ZnO nanowires were obtained after the introduction of a variety of reducing gases (H_2_, benzene, toluene) at 300 °C. Although the sensitivity of ZIF-8@ZnO was lower than the one for bare ZnO nanowires, its selective response towards H_2_ (kinetic diameter of 2.9 Å) over larger benzene (kinetic diameter 5.92 Å) and toluene (kinetic diameter 5.27 Å) gas molecules was highly improved. Similarly, Tian et al. produced ZIF-8@ZnO nanorods that were applied in the form of a paste onto an alumina-based substrate with Au electrodes and a heating resistor [58]. By following the conductimetric changes at 300 °C of the ZIF-8@ZnO sensor when formaldehyde was introduced into the testing chamber, linear concentration dependence between 10 ppm and 200 ppm was obtained. Despite that the response and recovery times were increased when compared to bare ZnO nanorods, the sensor showed no cross-sensitivity issues when exposed to water and different VOCs such as ethanol and ammonia, as well as acetone, methanol and toluene (Figure 6).

In some cases, selectivity does not exclusively depend on the size/shape exclusion effect, and it is rather determined by a mixture of polarity match and size compatibility with the MOF cages. For example, Davydovskaya et al. drop-coated Cu-BTC (BTC = 1,3,5-benzene-tricarboxylate; also known as HKUST-1) MOF on an Al_2_O_3_ substrate (with TiN as back electrode and Pt as heater) that served as a sensing element in a Kelvin probe setup for the detection of aldehydes [62]. Working function responses to pentanal were as high as 14.7 mV for a concentration of 10 ppm, whereas negligible responses were recorded for ethanal and propanal at 10 ppm and hexanal at 2 ppm. Pentanal responses were also measured in the presence of either ethanal or propanal with no remarkable changes in its work function. According to the authors, the Cu-BTC cages interact preferentially with non-polar molecules, which explain their selective response to pentanal. However, when a longer aldehyde like hexanal is used, the size-exclusion selectivity plays a role and may impede the penetration of bigger molecules. Similarly, Yamagiwa et al. coated quartz crystal microbalances (QCMs) and silicon-based microcantilevers sensors with either Cu-BTC or Zn_4_O(BDC)_3_ (BDC = 1,4-benzenedicarboxlate; also known as MOF-5) for the detection of VOCs [63]. The structural difference of the Cu-BTC and MOF-5 thin films, which were directly grown on COOH-terminated self-assembled monolayers (SAMs) onto gold electrodes of QCMs or silicon microcantilevers, was responsible for the selectivity towards different VOCs. The vapor response isotherms of a Cu-BTC-modified QCM towards ethanol and acetone, as well as toluene and *n*-octane, were compared to evaluate their dependence on the polar character and size of the analytes. The hydrophilic internal cages of Cu-BTC showed a preferential uptake for ethanol and acetone due to their polar nature. Moreover, the authors showed that no difference could be appreciated in the vapor response isotherms when alkanes and alkyl alcohols with identical chain length were used. However, the molecule sieving effect could be observed by increasing the chain length of both alkanes and alkyl alcohols with the following response sequence: *n*-hexane > *n*-hexanol > *n*-heptane > *n*-heptanol > *n*-octane > *n*-octanol. Additionally, the sensor displayed different responses to *o*-xylene, *m*-xylene, and *p*-xylene, which implies a certain differentiation between positional isomers. According to the authors, the lower selective adsorption of MOF-5 towards VOCs when compared to Cu-BTC was linked to its larger pore (0.8 nm) and aperture (1.2 nm and 1.5 nm) sizes.

### 3.2. Specific Adsorption Sites and other Effects

Selective chemical sensing can be achieved by taking advantage of the different functionalities found in MOFs nanopores. The host framework can selectively interact with the guest molecule to produce a specific signal. So far, the majority of works found in the literature that involve specific bonding interactions for gas sensing applications are based on luminescent MOFs [38,64,65,66,67]. 

**Figure 7:**
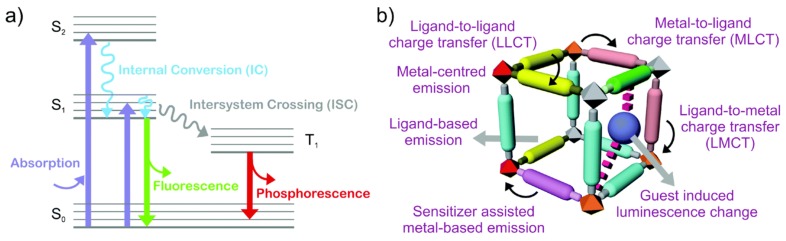
(**a**) Schematic illustration of different photo-physical processes, and (**b**) representation of the different options related to the optical emission of MOFs. Reprinted with permission from [71]. Copyright The Royal Society of Chemistry, 2017.

The sensing of gases by such optical means has gained popularity in recent years [50,67,68,69,70], and gas sensors based on an optical absorption or emission offer fast response times, high specificity with no cross-sensitivity, and longer lifetime than non-optical gas sensors. The characteristic emission of MOFs can be perturbed upon interaction with foreign guest molecules. Given the complexity of the different emission mechanisms, the assignment of an exact contribution to each of the ligand-metal interactions with guest molecules involved in the luminescence process is not trivial. Owing to their hybrid inorganic-organic nature, the luminescence of MOFs can be related to the linker or the metal ion. As the most common luminescence type, the luminescence emission from the linker can be further divided into ligand-to-metal charge transfer (LMCT) and metal-to-ligand charge transfer (MLCT). In addition, luminescence can also arise from the metals used as secondary building units (SBUs) or doped inside of the pores, as it occurs with lanthanide-based MOFs [71]. Figure 7 depicts the typical absorption and emission processes involved in the luminescence of MOFs.

**Figure 8:**
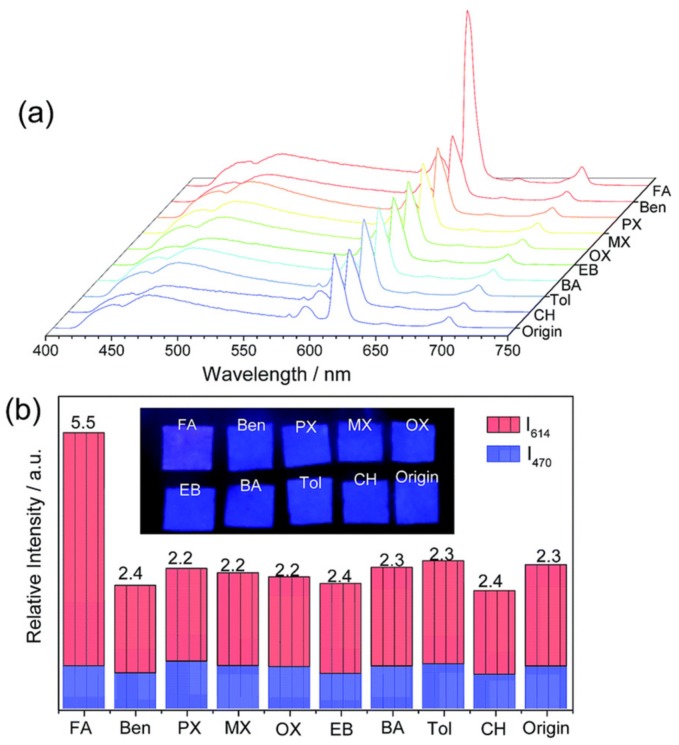
The PL spectra (**a**) and intensities (I614 and I470) (**b**) of Eu^3+^@ZUM test paper after exposure to different gases in cars (λ_ex_ = 365 nm). The photo insets of (**b**) are the corresponding pictures under the 365 nm UV irradiation and the given numbers are the I_614_/I_470_ values. Reprinted with permission from [38]. Copyright 2017 The Royal Society of Chemistry.

As a first example of this type of selective chemical sensing, Xu and Yan developed a Eu^3+^-functionalized ZnO@UIO-MOF (ZUM) for the sensing of aldehyde gases in vehicle exhausts [38]. The Eu^3+^@ZUM heterostructure overcomes some of the limitations of SMOs such as poor sensing performance towards concentrations at the ppb or sub-ppm level, and operation at high temperatures (usually higher than 200 °C) by using UiO-MOF as gas pre-concentrator, ZnO as reactive surface for aldehyde molecules, and Eu^3+^ as charge transfer element and fluorescent center. The gas sensing capabilities of Eu^3+^@ZUM were tested by impregnating a filter paper in an ethanolic solution of Eu^3+^@ZUM and exposing it to a variety of volatile gases such as benzene, toluene and xylene, but also formaldehyde and cyclohexane, with a concentration of 10 ppm at 25 °C during 30 min while following their photoluminescence (PL) emission spectra (Figure 8). Except for formaldehyde, none of the other volatile gases presented an increase in Eu^3+^ based PL emission, and a change of color of the testing paper from blue towards red was perceived by the experimenter’s eye. In addition, concentration-dependent PL measurements to formaldehyde were carried out and a limit of detection of 42 ppb was estimated. Additionally, when exposed to a mixture of formaldehyde (10 ppm) and other interfering VOCs (30 ppm), the Eu^3+^@ZUM test paper gave the same response as when exposed to formaldehyde alone, which indicates no cross-sensitivity to other VOCs. The sensor was exposed to acetaldehyde and acrylaldehyde to test the response of the composite material to other aldehydes. As in the case of formaldehyde, the sensor shows a response to acetaldehyde and acraldehyde with limits of detection of 58 ppb and 66 ppb, respectively. The Eu^3+^-functionalized ZnO@UIO-MOF showed promising sensing properties towards pollutant aldehydes in VOCs mixtures and the possibility of being reused by being tested sequentially during five cycles.

**Figure 9:**
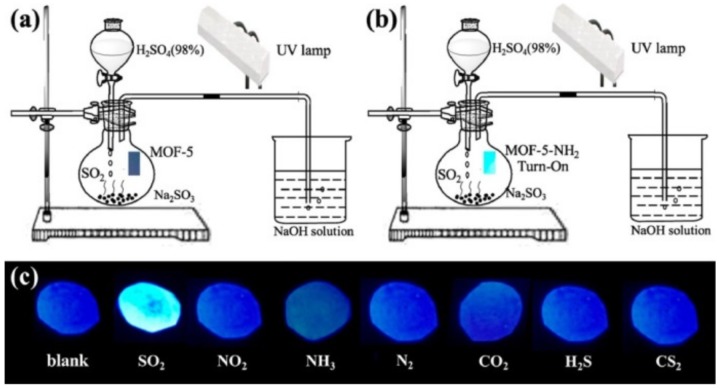
(**a**,**b**) Diagrams of the sensor for the detection of SO_2_ gas using MOF-5 and MOF-5-NH_2_ luminescent testing paper, and (**c**) luminescence response photos of MOF-5-NH_2_ test paper after exposure to different species under a (365 nm) UV lamp [66]. Copyright American Chemical Society, 2018.

Similarly, Hao and Yan developed a fluorescent sensor based on a Eu^3+^ postfunctionalized MOF for the ammonia detection [65]. The 3D open framework with rhombic pores and 2,2-bipyridine accessible sites was used to coordinate Eu^3+^ cations. The gas sensing element was produced by spin coating an ethanolic solution of Eu^3+^@Ga(OH)bpydc on a quartz substrate to produce a luminescent thin film. The selectivity towards ammonia was confirmed by testing the sensor in the presence of several indoor gas pollutants (formaldehyde, benzene, toluene, *o*-xylene, ethylbenzene, styrene, butyl acetate, and *n*-undecane). All of the organic vapors tested had an insignificant effect on the luminescence of the MOF film except for ammonia, which provoked a quenching effect (about 76%) on the emission intensity of Eu^3+^ at 614 nm and could be observed by the naked eye. Moreover, after the sensing film was exposed to a mixture of ammonia and the rest of the organic vapors, the emission spectra displayed a similar signal at 614 nm to that of the ammonia only. These results prove the excellent selectivity of the MOF sensor. In a similar approach, Wang et al. fabricated a luminescent sensor based on amino-modified MOF-5 for the detection of SO_2_ [66]. To enhance the portability of the sensor, blank neutral paper was dipped into a suspension of MOF-5-NH_2_. When exposed to a series of gases (SO_2_, NO_2_, NH_3_, N_2_, CO_2_, H_2_S, and CS_2_), the test paper only displayed a turn-on effect for SO_2_ (Figure 9). According to the authors, SO_3_^2-^ (since SO_2_ and SO_3_^2−^ can lead to a gas-liquid equilibrium) anions to form complexes when interacting with the amino group that inhibit the electron transfer between NH_2_ and coordinated metal ions (LMCT), thus resulting in a luminescence turn-on effect. In addition, the test paper was exposed to a set of concentrations (0.1, 0.5, 1, 2, and 3 ppm) of SO_2_ gas and an increase of the luminescence intensity was observed with increasing SO_2_ concentration. 

It is widely recognized that changes in the metal coordination sphere [72], anion driven structural transformation [73], and guest inclusion [44] can result in a color change that can be appreciated visually. Zhao et al. reported the fabrication of a Cu_4_I_4_-MOF that was used as a highly selective naked eye sensor for HCl in the gas phase [74]. The Cu_4_I_4_-MOF was prepared as light yellow crystals by combining a ligand bearing 3-pyridil group and CuI in CH_3_CN. When exposed to HCl, a color change of the crystals from orange to dark brown was observed and remained visible in the concentration range of 2.4–3.2 ppm within 30 min. The authors explained the visual color change with a scheme that involves the selective replacement of I^−^ ions on the nodes with Cl^−^ and an immediate oxidization of I^−^ to I_2_ in the presence of oxygen from air and H^+^. Furthermore, the Cu_4_I_4_-MOF showed an improved sensitivity towards HCl (0.8–1.6 ppb) by observing variations in its emission spectra. Additionally, an almost complete quenching of the emission was observed at 8 ppb. The selectivity of the sensor was examined by exposing it to a series of analogue gases (HF, HBr, HI, HOAc, and HClO_4_). No color or emission spectrum changes could be observed, even at high concentrations for the analogue gases. Additionally, the selectivity towards HCl in a mixture with the analogue gases was confirmed by obtaining a similar response as the one for pure HCl. 

**Figure 10:**
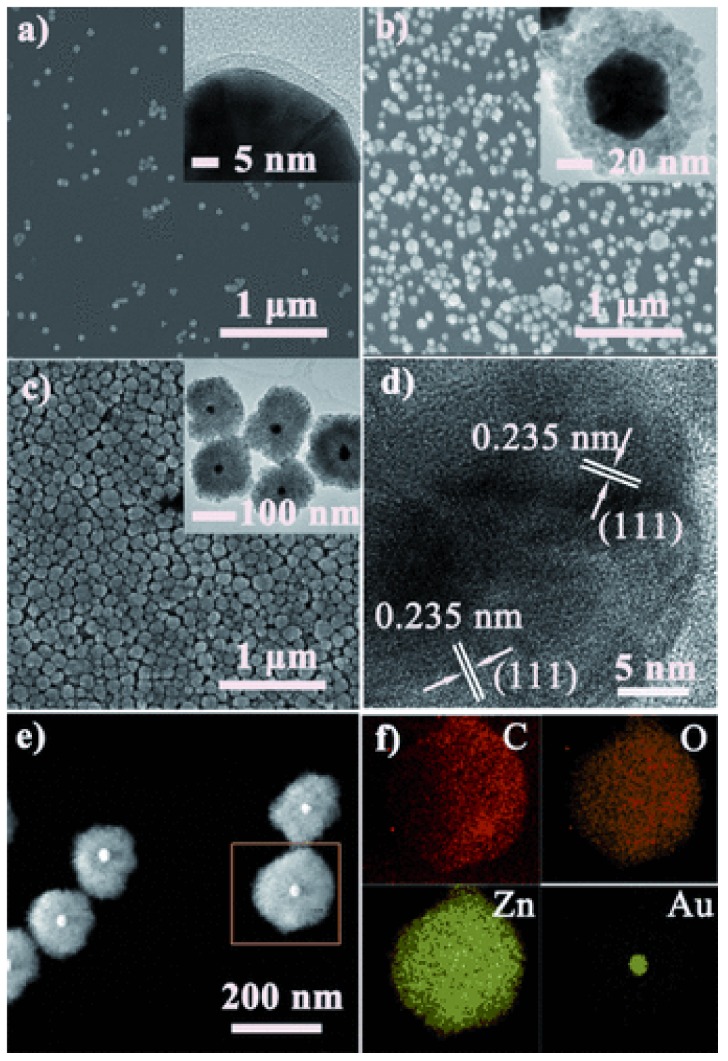
SEM and TEM (insets) images of the core–shell Au@MOF-5 NPs presenting shell thicknesses of (**a**) 3.2 ± 0.5 nm, (**b**) 25.1 ± 4.1 nm, (**c**) 69.0 ± 12.4 nm, (**d**) TEM image of a core–shell Au@MOF-5 NP sample, (**e**) HAADF-STEM image of Au@MOF-5 NPs presenting a shell thickness of 69.0 ± 12.4 nm, and (**f**) EDX mapping of the Au@MOF-5 NP marked in (**e**). Reprinted with permission from [77]. Copyright John Wiley and Sons, 2013.

**Figure 11:**
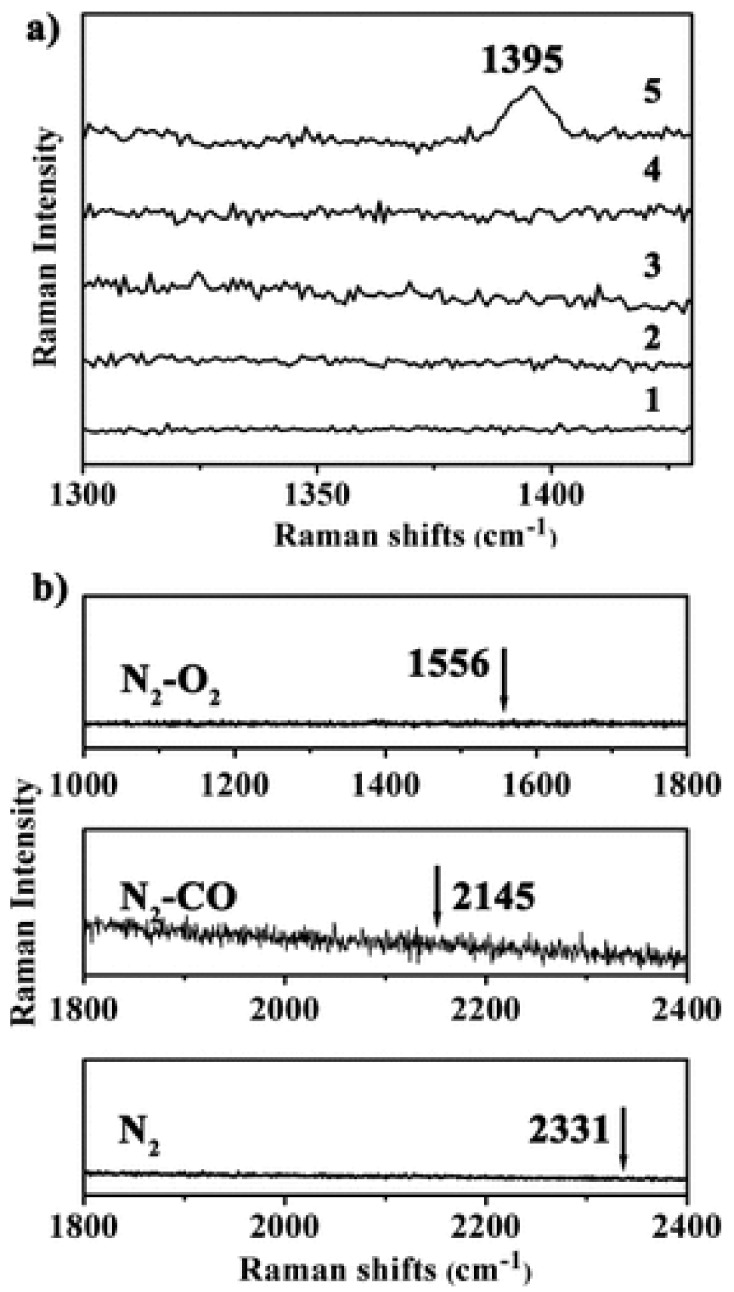
(**a**) SERS of single Au NPs (trace 1), of single MOF-5 (trace 2), and single core–shell Au@MOF-5 NPs presenting shell thickness of 69.0 ± 12.4 nm (trace 3), 25.1 ± 4.1 nm (trace 4), and 3.2 ± 0.5 nm (trace 5) towards CO_2_ in the CO_2_/N_2_ mixture, and (**b**) SERS spectra of Au@MOF-5 NP with a shell thickness of 3.2 ± 0.5 nm towards N_2_, CO, and O_2_. The characteristic SERS peaks of N_2_, CO, and O_2_ are shown by arrows. Reprinted with permission from [77]. Copyright John Wiley and Sons, 2013.

The vast literature on MOFs employed as materials for gas separation and storage can offer a hint about the structures and functionalities that might be useful for gas sensing [12,75]. In particular, the electrostatic interaction between adsorbates and MOFs has been proposed as a key mechanism that can be exploited for CO_2_ selectivity in gas separation processes [76]. For instance, He et al. fabricated core-shell Au@MOF-5 nanoparticles for selective detection of CO_2_ in gas mixtures [77]. Au@MOF-5 nanoparticles with three different shell thicknesses (~3 nm, ~25 nm, and ~69 nm) were synthesized by mixing Au and MOF precursors in a one-pot synthesis fashion while controlling the reaction conditions (Figure 10). Owing to the inherent plasmonic activity of Au and the affinity of CO_2_ for MOF-5, the surface-enhanced Raman scattering (SERS) performance of the core-shell Au@MOF-5 nanoparticles was investigated by measuring their Raman spectra in a CO_2_/N_2_ gas mixture. While no Raman signals could be identified from bare Au nanoparticles, MOF-5 spheres, core-shell Au@MOF-5 ~25 nm, or Au@MOF-5 ~69 nm, the ~3 nm Au@MOF-5 nanoparticles displayed a characteristic CO_2_ Raman peak at 1395 cm^−1^ (Figure 11).

Considering that the electromagnetic enhancement coming from Au is attenuated exponentially with distance, the shell of the ~3 nm Au@MOF-5 nanoparticles is thin enough to selectively capture CO_2_ while boosting the Raman intensity by a 2.4 × 10^3^ factor. Additional SERS measurements were conducted in the presence of N_2_, CO, and O_2_ and no SERS activity was observed. This highly selective behavior was explained by the electrostatic attraction between CO_2_ molecules and the aromatic rings from MOF-5.

## 4. Nanomembrane Layers Prepared by Atomic Layer Deposition (ALD)

### 4.1. Introduction to ALD

A key strategy for the direct growth of thin films with controllable dimensions at the nanometer scale is atomic layer deposition (ALD). ALD is based on sequential and self-limiting chemical reactions, allowing the preparation of inorganic nanomaterials such as oxides [13,14], nitrides [18,19,78] and metals [22,23], with a (sub)nanometer thickness control [24,25]. Typically, during an ALD cycle, alternate pulses of precursors and co-reactant gas molecules are injected in a vacuum reactor, separated by purge/pumping steps [24,79]. The advantages of the ALD technique are thickness control at the atomic-level, the excellent uniformity and the conformality over the substrate surface. This route is therefore an excellent technique to coat complex 3D substrates such as nanowires and other nanostructures [24,25,80,81,82]. These key benefits allowed this technology to become a key route for the deposition of thin films for a wide range of applications, especially microelectronics [83,84], but the technique is also used for catalysis [85,86], membranes [19,87,88], photovoltaics [89] and sensing [90]. 

### 4.2. ALD Layers for the Enhancement of Gas Sensors

**Figure 12:**
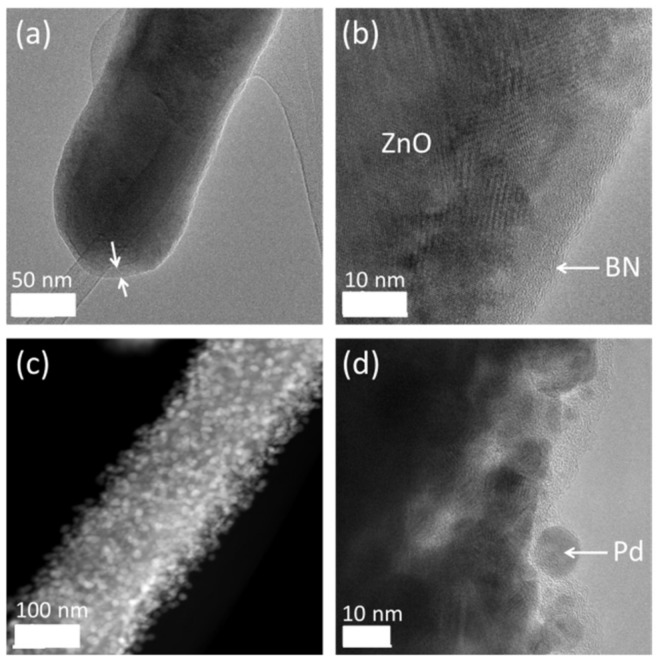
TEM images of (**a**,**b**) the BN/ZnO NWs and (**c**,**d**) the Pd NPs/BN/ZnO NWs. In (**a**) and (**b**), the BN layer is shown by arrows. In (**d**), a Pd NP is designated. Reproduced from [39] with permission from The Royal Society of Chemistry, 2019.

Semiconductor metal oxide materials are used as gas sensors because of their chemoresistant behavior. The incorporation of the gas sensitive material in a sensor as a thin film is very advantageous, as it makes the sensor production compatible with semiconductor manufacturing processes, allows for miniaturization and relatively low costs. Furthermore, a thin sensing layer results typically in high sensitivity, fast response, and minimal power consumption. Therefore, ALD has been used for the fabrication of various gas sensing materials. For example, Du and George synthesized SnO_2_ films for CO sensing [91], and Aronniemi et al. applied ALD for the production of iron oxide films applied to CO and O_2_ sensing [92]. Other gas sensing semiconductor compounds have been fabricated by ALD such as TiO_2_ [93], ZnO [94], or In_2_O_3_ [95] for examples. Recently, our group reported a novel route for the preparation of gas sensors with enhanced hydrogen selectivity, by using ultrathin BN layers prepared by ALD [39]. The sensors presented in this study were based on ZnO nanowires coated with a 5 nm BN film prepared by ALD decorated with Pd NPs. Figure 12 shows TEM images of these nanowires.

Due to the difference in work function between ZnO and the BN monolayer, electrons from Schottky barriers are formed on the interfaces which expand the electron depletion layer, when compared to pristine ZnO NWs. Upon exposure of the gas sensor to dihydrogen gas and release of electrons to ZnO NWs surface, the barrier height is decreased, leading to a higher modulation of the resistance and thus a higher sensor response. Furthermore, as Pd can efficiently adsorb and dissociate H_2_ molecules upon exposure to dihydrogen gas, the metallic Pd may be partially converted to PdH_x_. Due to the difference in the resistance of Pd and PdH_x_, this conversion is also expected to increase the gas sensor sensitivity [39]. Finally, due to the BN structure which is somewhat similar to graphene [96,97], the ultrathin film can act as an efficient nanomembrane and let hydrogen atoms reach the surface of the ZnO NWs forming the transducer of the sensor. Due to their very small size, hydrogen atoms could pass the BN layer, whereas the other molecules tested such as C_6_H_6_, C_7_H_8_, C_2_H_6_O and C_3_H_6_O could not cross this layer. This phenomenon enabled to considerably improve the sensor selectivity towards hydrogen [39]. 

Similarly, Mirzaei et al. prepared ZnO layers by ALD to cap Fe_2_O_3_ nanorods based gas sensors. The sensing characteristics of the core/shell nanorods for ethanol were studied [98]. The increased response of the Fe_2_O_3_–ZnO nanorods sensor are linked to the modulation of the width of the conduction channel and the potential barrier height at the level of the interface, as well as to a nanomembrane effect of the ZnO coating. In fact, crystallographic defects are formed at the Fe_2_O_3_–ZnO interface resulting from the lattice mismatch between the two nanomaterials, providing preferential adsorption sites and paths for small molecules such as oxygen and ethanol, also contributing to the increased ethanol sensing properties of the core-shell structured nanorod sensor [98].

Although this is not typically desired and strongly depends on the process used, ALD films can present a residual porosity. Using ellipsometric porosimetry, Perrotta et al. extensively studied the residual nanoporosity (0.3–2 nm) in alumina and SiO_2_ layers fabricated by (plasma-assisted) ALD and its role in controlling the oxygen and moisture barrier performance [99,100,101]. A correlation between the nanoporosity of the films and their intrinsic barrier properties has been observed, regardless of the film chemistry [99]. In fact, pores presenting diameters higher than 1 nm with a relative content above 1% have been found responsible for the low barrier performances obtained.

When taking into account this residual nanoporosity, thin films prepared by ALD can advantageously be employed as selective nanomembranes to coat functional materials, for different purposes such as catalysis and gas sensing. Thus, ALD nanolayers can also be used to enhance the selectivity using the molecular sieving effect.

**Figure 13:**
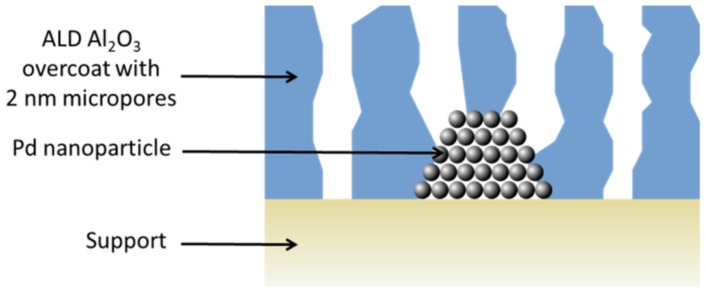
Schematic representation of a supported Pd nanocatalyst with a thermally activated ALD Al_2_O_3_ microporous overcoat. Adapted from [102], American Association for the Advancement of Science, 2012.

For example, it has been shown that ultrathin alumina overcoats (8 nm) over supported metal nanoparticles could be used as nanomembranes (after thermal annealing activation), successfully enabling the oxidative dehydrogenation of the ethane molecules crossing the micropores of the alumina, meanwhile reducing the deactivation by coking and sintering at high temperature [102]. Figure 13 depicts the Pd/Al_2_O_3_ catalysts with the thermally activated ALD Al_2_O_3_ overcoat. 

Similarly, Wang et al. coated palladium nanocatalysts with Al_2_O_3_ as well as FeO_x_ ultrathin films and measured their catalytic properties. Again, they observed a remarkable (benzaldehyde) selectivity for the overcoated 4-nm Pd nanocatalysts, depicting the efficient use of the ALD layer nanomembrane to separate different molecules [103]. Again, these examples clearly show the potential use of ALD nanolayers towards the enhancement of selectivity, using the molecular sieving effect.

### 4.3. Perspectives of Other ALD Materials

**Figure 14:**
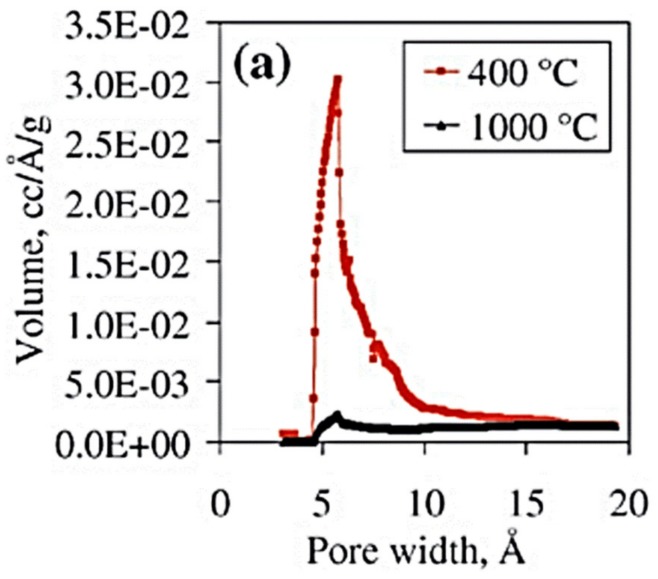
Pore size distribution of the micropores formed on a 25 nm film of MLD alucone after oxidation at 400 °C and 1000 °C in air. The substrate was SiO_2_ particles. Reprinted with permission from [109], Royal Society of Chemistry, 2009.

Since ALD can be described as a CVD-derived method, molecular layer deposition (MLD) can be seen as an ALD-derived method [104]. MLD is in fact also based on sequential and self-limiting surface reactions, and this route is very close to ALD, the main difference being that organic compounds are used instead of the classic (metal-organic) ALD precursors or reactants. The resulting films prepared by MLD are either hybrid organic–inorganic films or purely organic [104]. Although many (“metalcone”) materials can be prepared using MLD, alucone is the most commonly synthesized, with a process typically based on trimethylaluminium (TMA) molecule as precursor and ethylene glycol as co-reactant [105,106]. As etching and calcination post treatments can be applied to remove the organic component from the prepared film and leave a porous inorganic matrix, the organic–inorganic MLD “metalcone” films can be converted into porous (inorganic) thin films [107,108]. The conformality of the MLD prepared films and their transformation into porous ultrathin layers enable the synthesis of porous oxide nanomembranes, with controllable thickness and porosity. For example, for alucone films prepared with a TMA and ethylene glycol process, the micropore size is centered at 0.6 nm, which is due to the removal of the monolayer of (–CH_2_CH_2_–) groups. The resulting microporous monolayer obtained could advantageously be applied to enhance the selectivity of sensors or other devices [107]. Figure 14 shows the distributions of pore sizes of micropores formed on a 25 nm of alucone after oxidation at 400 °C and 1000 °C (in air) [109]. 

The thickness of the nanomaterials can be tuned by varying the number of MLD cycles, and the size of the pores can be controlled by tuning other process parameters [109]. The length of the organic chains of the organic co-reactant chosen has the main influence on the pore size [108,109], but other parameters involved in the etching or calcination treatment can be tuned as well. For example, dependence between the porosity of the films and the applied heating ramp rate has been reported [109]. Therefore, many highly porous oxide films with tunable thickness have been prepared by thermally treating MLD films, and such oxide nanostructures can present surface areas as high as 1250 m^2^/g [108,109]. For example, by removing the bridging organic template, Jiang et al. have successfully synthesized ultrathin nanomembranes on porous supports for gas separation. Due to the thinness of the nanomembranes, after 200 cycles, the He permeance was as high as 5.3 cm^3^/bar·cm^2^·min, and the selectivity of He/N_2_ and He/SF_6_ exceeded 10^3^ and 10^4^, respectively [110]. By overcoating zeolite membranes with microporous MLD membranes, Yu et al. achieved the preparation of microporous and defect-free Al_2_O_3_/SAPO-34 zeolite composite membranes. These inorganic membranes present a very high H_2_/N_2_ mixture selectivity of 1040, in contrast with selectivity of 8 for the uncoated SAPO-34 membranes [111]. As these highly selective porous layers can be deposited in a conformal manner, they could efficiently be used for the enhancement of the selectivity of gas sensing nanostructures.

In addition, the ALD method has also been developed for the tuning of MOFs materials and even the preparation of MOF thin films in the recent years. For example, vapor phase metalation in an MOF can be precisely carried out by ALD [112], enhancing the properties of the hybrid nanomaterial. Furthermore, the self-limiting growth of MOF-5 thin films was achieved by the group at Helsinki University [113], and the thin film deposition of the thermally and chemically stable UiO-66 in an all-gas-phase process by ALD has been reported as well [114]. Thus, the advantages of ALD, such as excellent conformality, can be exploited for the synthesis of selective MOF coatings as well.

If the main application reported is the sensing of small molecules such as hydrogen gas, one should note that the chemical compatibility or the temperature stability of the nanomaterials employed (MOFs and ALD based) have to be considered, and can represent a limitation to their practical use.

Other innovative and more stable materials that can be prepared by ALD and used to improve the selectivity of gas sensors include the so-called “2D materials” [115,116]. 2D nanostructures can be made of different materials such as metal oxides, graphene, dichalcogenides, phosphorene, BN and MXenes, and typically present thicknesses that may fluctuate from a few to tens of nanometers and lateral dimension of up to many centimeters [117]. In fact, in the recent past, these 2D materials have been the focus of interest for many potential applications, including gas sensing [118,119]. The gas sensing function of 2D materials such as graphene is related to a direct charge transfer mechanism, relatively different from metal oxides [120,121]. In fact, the adsorption of gas molecules will modify the graphene charge carrier concentration by either decreasing or increasing the electron concentration, depending on the gas molecules (electron acceptor or donor), which result in an increase or decrease of the electrical conductivity (sensor response) [120,121].

## 5. Conclusions

In this work, the basics on the mechanisms and configuration of gas sensors have been presented, and the application of MOFs materials and ALD films as nanomembranes towards the separation of gas molecules and the enhancement of gas sensor selectivity have been illustrated. Among the possible mechanisms of gas selectivity enhancement, the most used is the size-exclusion effect (molecular sieving) using porous overcoats, where only analytes presenting dimensions smaller than the pores can go through, while larger molecules cannot. 

In the case of MOFs, the size as well as the aperture of the pores can be fine-tuned by selecting the appropriate metal ion clusters and organic linkers. Furthermore, the selectivity of MOFs nanomaterials towards specific molecules can occur through chemical functionalities, which can be achieved by incorporating the desired functionality directly within the MOFs nanostructure during their synthesis. One ultimate goal to enhance the use of MOFs to enhance their selectivity would be to precisely control their orientation, which would allow for a high and selective flux.

ALD nanomaterials have been used to enhance the sensitivity and the selectivity of gas sensing devices. For example, specific supports and catalysts can be prepared conformally on top of sensors to improve their sensitivity. In addition, overcoating a device with an ALD thin film and thermally activating it can convert it into a microporous and highly selective nanomembrane. Similarly, MLD prepared films and their transformation into porous layers enable the synthesis of conformal and porous layers with controllable porosity, opening prospects for their use to enhance the selectivity of gas sensing devices.

This review comprehensively showed how MOFs and ALD nanolayers can be and could be used towards the enhancement of selectivity of gas sensing devices, opening up prospects for the sensors community.
